# The Future of School Medical Inspection

**Published:** 1916-10-21

**Authors:** 


### THE FUTURE OF SCHOOL MEDICAL INSPECTION.

### By a School Medical Officer.

Of all the lessons which the present war has taught us, one of the most outstanding is the paramount importance of maintaining and safeguarding the health of the rising generation if we are to preserve that position amongst the nations which enables us not only to resist for ourselves the menace of invasion, but also to protect the smaller States from the brutal aggression of a ruthless enemy. Of measures for the protection of the child's health the only one which was at all organised by the State before the war was that of school medical inspection. The war has proved, if any proof were necessary, how vital this inspection is in the prevention of defects which in after-life may render the grown man unfit to be an asset to his country, and may also cause him or her to be a drain on the financial resources of the community at a time when they are most needed for other purposes.

It is during the growing years that the foundations of a healthy and long life are laid, or the seeds of disease are sown, which may ultimately cut off the individual in his prime, or cause him to be unfit for any service to the State. Many of the defects which now cause a recruiting medical officer to reject a man, or show themselves during training, might easily have been prevented if the causes leading to them had been recognised and corrected during childhood. The necessity for the continuance and growth of medical inspection of school children has, therefore, more than ever been proved by the war, and every effort should be made to improve its efficiency and widen its scope in the future.

### Defects which are Overlooked.

The minimum of inspection and treatment is, unfortunately, all that can be carried on while the war continues, as no fewer than 300 out of 850 school medical officers have joined the Forces. Of these we will hope that the majority will return to this work; the positions of most are being kept open for them by the local education authorities. Some, alas! will have given their lives or health in the service of their country, and others may find that the wide outlook opened for them by new activities in other countries may render their former work too tame an exercise for their energies. All of them, however, who return must realise more than ever the importance of the work which they are called upon to perform, after having seen how the defects which are most easily corrected in childhood appear in the adult, and render him unfit to serve his country, or cause him to break down when he is put to any strain such as that of military service. It will teach them also to look particularly for defects to which they, perhaps, hitherto have not paid sufficient attention, especially, to take only one instance, that of a tendency to malformation of the feet, and to take measures for their correction. If statistics of causes of rejection of recruits, and discharges of men found unfit during training, were taken it would probably be found that defects of the feet and legs were responsible for almost or quite half, and such defects are just those which could have been prevented during childhood by timely advice to the parents of the child.

Growing out of school medical inspection, schools for mothers, pre-natal clinics, etc., have arisen, and this work will and should grow in future as the child has no special measures for the protection of his health provided by the State before he enters school. Further, the school age for entrance has recently been raised to five years on account of the exigencies of the war and the consequent depletion of the teaching staffs. Although this age will probably be lowered after the war it is of great importance that the child should have some State medical supervision during babyhood. Whether these pre-school health measures can be co-ordinated with school medical inspection and carried out by school medical inspectors is a point yet to be realised; it may be possible in towns, but it is difficult to see how it can be done in the country, where the population is scattered over many villages. Whether it can be carried out by school medical inspectors, however, in addition to their other work, or is relegated to a special staff of private practitioners or others, it is certain that close co-ordination of the two should be maintained, and that the school medical inspector should have the opportunity of referring to the previous medical history of every child whom he is called upon to examine.

### Continuous Health Supervision.

In this way a continuous chain of health supervision could be kept up during the life of every individual of the proletariat from pre-natal days to death by: (1) pre-natal clinics and schools for mothers, (2) health supervision of children before school age, (3) school medical inspection, and (4) during adult life, to some extent, by the medical provisions of the National Insurance Acts. Of course a system of this sort would present many difficulties and take a considerable time before it could be organised, but the trouble and expense involved would be amply repaid by the resulting preservation of life, and the increased happiness and well-being of the community at large. A proposal of a full scheme of health-supervision by the State such as this would be sure, also, to be met by the objection that it would lead to a further poaching on the preserves of the long-suffering general practitioner. After the war, however, it is certain that the profession will have to undergo a process of reorganisation which, let us hope, will render the position of the general practitioner more secure, without interfering with any further health measures which may be necessary for the well-being of the community.

